# Supplementary material for: Getting More Out of Biomedical Documents with GATE's Full Lifecycle Open Source Text Analytics
Source: PLoS Comput Biol. 2013 Feb 7;9(2):e1002854. doi: 10.1371/journal.pcbi.1002854 (PMC3567135; doi:10.1371/journal.pcbi.1002854)
Supplement: Dataset S1 — GATE software. Dataset S1 bundled with this paper contains a distribution of GATE (or see http://gate.ac.uk/download/). (TGZ) [file pcbi.1002854.s001.tgz › plos-gate/plugins/Lang_Arabic/resources/inferred-gazetteer/BBN-Types-Subtypes.doc]

ANNOTATION GUIDELINES FOR ANSWER TYPES
BBN TECHNOLOGIES
Ada Brunstein
8/03/02
© Copyright BBN Technologies 2002

Below are annotation categories and guidelines assembled by BBN for the Question Answering task.  The 29 types represent answer categories and are described in the table below.  Following the table are brief annotation guidelines.   

The description column in the table includes a general description of a category, some examples, and the sgml string that was used in annotation.  For types that have subtypes, the subtype column includes examples of each of the subtypes for that particular type and shows only the markable entity for that type and subtype in <>.  The SGML string for that subtype is also given.  Most examples come from the WSJ Treebank data.  

These 29 categories include the five EDT types, Person, Organization, Location, GPE, Facility, with some minor modification to those guidelines.  For example, under EDT guidelines museums are facilities whereas here they are organizations.  They also include the MUC categories Money, Percent, Time and Date, again with some modifications.  For example, in the classification below Date includes durations, dates (relative and absolute) and age, each of which is identified as a subtype in the annotation.   

The remaining categories were determined in part based on categories found in literature on the question answering task and in part based on BBN’s own examination of the data.


BBN’s PROPOSED ANSWER CATEGORIES FOR QUESTION ANSWERING

Type
Brief Description
Subtypes

Person 
Name
Markable:  Proper names of people, including the following: fictional people, first names, last names, individual or family names, unique nicknames.  Generational markers such as Jr. and IV, are included (Ray Garrett Jr., Henry IV). 

SGML:  <ENAMEX TYPE="PERSON">Michael Henderson</ENAMEX>
No Subtypes

Person 
Descriptor


Head of Descriptor
Markable:  Any head word of a common noun referring to a person or group of people.  Any occupational title in modifier position.  For example, for ‘President Bush’ we have a descriptor (‘president’) and a name (‘Bush’).   

Unmarkable:  Honorific titles such as Mr., Mrs., Sir, etc.

Examples:  (note, only descriptor annotation is indicated)
	his top <aides> 
	die-hard <fans>
	<analysts> said
	State court <Judge>  Richard Curry
	The <owner> , who begs anonymity
	<Chief Executive>  Sir   Christopher   Hogg
	<Chairman> Jay B. Langner

SGML:   <ENAMEX TYPE="PER_DESC"> </ENAMEX>
No Subtypes

NORP

Name 
This type is named after its subtypes, nationality, other, religion, political.  The distinction between NORP and other types is morphological.  American and Americans is a nationality, while America and US are GPEs, regardless of context.

Markable:  Adjectival forms of GPE names and Location names (such as American).  Adjectival forms of named religions, heritage and political affiliation (such as Democratic, Chinese-American, and Jewish).  Head words which refer to people using the name of an entity they are affiliated with, often GPE, Location, or Organization.  For example, the three Democrats, the Muslims, etc.   [See subtypes for more examples.]


Nationality: Adjectival form of GPE/Location modifier,  (an American, Chinese soil, European organization, etc.) or head word referring to a person by the name of the GPE or Location they are from.

	the <Brazilian> development
	<European> euphoria
	twice as many  Nobel  science prizes as the <Japanese>
	the <Dutch> publishing group
	a fellow <Texan>
	a conservative <Californian>
	<Irish-American>  (note that this is marked as one entity, not two)

Religion: Any adjectival form of a religion, regardless of context.

	Mr. Sohmer , who is <Jewish>
	a devout <Catholic>
	The <Hindu> newspaper

Political: 
	Some <Democrats>
	a <Communist> official
	France's <Socialist> government

Other:	
	<African-American> activist
	<Arab> uprising
	<Francophone>
	< Mafioso>

Facility Name
Names of man-made structures, including infrastructure, buildings, monuments, etc.
See subtypes for examples.
Building: buildings, cathedrals, laboratories, monasteries, plants, refineries, stadiums, synagogues, towers, vineyards, etc.

Bridge: bridges
	<G Street Bridge>
	<Golden Gate>

Airport: airports, parts of an airport including terminals, runways, and towers, air force bases

Highway_Street: avenues, boardwalks, highways, plazas, streets, exits
	<Interstate 64>
	<Madison Avenue>
	<Exit 10>

Attraction: amusement parks, tourist attractions, aquariums
	<Statue of Liberty>

Other:  courtyards,  farms,  (gas) stations, Space Centers, (Labor) camps, mines, oil fields, ports, (subway) stations, golf courses


Facility 
Descriptor

Head of 
Descriptor
Head noun of a noun phrase describing buildings, bridges, airports, highways, attractions…
(Same categories as for Facility Name, but with different examples)

Building: 
	the <plant> 's construction cost
	spending on single-family <homes>
	built in a <garage>
	a new computer <lab>
	The entire second <floor>
	a third <room>
	Westinghouse would own 70 % of the <facility>

Bridge: bridges
	the collapsed Route 880 <overpass>
	Roads and <bridges> in the Bay area

Airport: airports, parts of an airport including terminals, runways, and towers, air force bases
	build a cargo <terminal>
	travelers stuck at some <airports>

Highway_Street: avenues, boardwalks, highways, plazas, streets, exits
	looking for an <interstate>
	tiny shops and twisted <alleys>
	traffic <intersections>

Attraction: amusement parks, tourist attractions, aquariums
	1.5 million-gallon saltwater <aquarium>

Other:  courtyards,  farms,  (gas) stations, Space Centers, (Labor) camps, mines, oil fields, parks, ports, (subway) stations, 
	space <station>
	six <mines>
	<junkyard>
	The <dam>


Organization 
Name
Markable:  Names of companies, government agencies, educational institutions and other institutions:  sport teams, hospitals, museums, and libraries.  White House, Pentagon, Kremlin are ORG names as is Capitol Hill.  Adjectival forms of organization names are also included:  Congressional efforts, etc.  

Newspaper names should be marked as ORG names regardless of whether they refer to the artifact or the organization.  

Organization names also include products that are referred to by their manufacturer’s name (as in “I bought a Buick”).  

Annotators will have to determine whether groups, teams, forces, etc. refer to people or organizations.

Unmarked: organized crime
Government: 
Names: government organizations (State Department, SEC, military, etc.), American [FBI] – nationality before an abbreviation is marked separately, Attorney General’s Office, Capitol Hill, City Hall, European Union, EU, European Community, EC, Interpol, Minfin, NATO, NYPD, OPEC, Parliament, Prosecutor's Office, Russian Interior Ministry / Russian ministry of the interior, State Duma, United Nations, UNESCO, US Army, US Embassy 

Descriptors: GPE governments, administration (i.e. Clinton/US administration), jury, militia, police department – only “department” is marked when the Dept name is not given, Regulators, state - when used to refer to the gov’t (if it refers to the State Department then it is marked ORG Name.

Corporation: Big Board, boards of private organizations, casinos, daily/weekly if used to describe a newspaper or magazine, divisions of private organizations, media organizations (TV, radio, newspapers, magazines), private organizations, shops, stock exchanges, sometimes ventures 
		
SGML: <ENAMEX TYPE="ORGANIZATION:CORPORATION"> </ENAMEX>

Educational: schools, universities

SGML: <ENAMEX TYPE="ORG_DESC:EDUCATIONAL">  </ENAMEX>

Religious: churches, mosques, etc.

SGML:  <ENAMEX TYPE="ORGANIZATION:RELIGIOUS"> </ENAMEX>

Political: political parties: Contra(s), Democratic Party, PLO, Sandanista, Taliban	

SGML:  <ENAMEX TYPE="ORGANIZATION:POLITICAL">  </ENAMEX>

Museum: museums

SGML:  <ENAMEX TYPE="ORGANIZATION:MUSEUM"> </ENAMEX>

Hotel: hotels, resorts

SGML:  <ENAMEX TYPE="ORGANIZATION:HOTEL">  </ENAMEX>

Hospital: hospitals

SGML:  <ENAMEX TYPE="ORGANIZATION:HOSPITAL">   </ENAMEX>

Other: libraries, unions, environmental agencies, professional associations, health associations (Planned Parenthood), musical groups

Names: city or country name refering to a sports team, i.e. “<Boston> shortstop Nomar Garciapara”, IMF, National Sports Fund, Planned Parenthood, Professional Organizations (Health Ins. Association of America, etc.), Red Sox, (city names preceding teams are considered part of the team name, as in New York Yankees), World Bank, Yankees

SGML:  <ENAMEX TYPE="ORGANIZATION:OTHER">  </ENAMEX>


Organization Descriptor

Head of Descriptor
Markable:  Heads of descriptors of companies, governments, government agencies, educational institutions and other institutions:  sport teams, hospitals, museums, and libraries.  In “I bought the newspaper”, “the newspaper” is not an organization descriptor so it is unmarkable.  References to “offices” should be marked organization descriptor.

SGML:  <ENAMEX TYPE="ORG_DESC:SUBTYPE">  </ENAMEX>
See Organization name for subtypes

GPE 
Name
Markable: Names of countries, cities, states, provinces, municipalities.  In cases like “southern California”, California” is marked as a GPE name and there is no other markup.
Country: countries, nations

SGML:  <ENAMEX TYPE="GPE_DESC:COUNTRY">  </ENAMEX>

City: cities, towns, villages, Washington & DC are tagged separately

SGML:  <ENAMEX TYPE="GPE:CITY">  </ENAMEX>

State/province: states, provinces

SGML:  <ENAMEX TYPE="GPE:STATE_PROVINCE"> </ENAMEX>

Other: Boroughs, islands that have governments

SGML:  <ENAMEX TYPE="GPE:OTHER">  </ENAMEX>

GPE Descriptor

Head of Descriptor
Markable: Heads of descriptors of countries, cities, states, provinces, municipalities

SGML:  <ENAMEX TYPE="GPE_DESC:SUBTYPE">  </ENAMEX>
See GPE Name for subtypes.

Location
Name
Markable: Names of locations other than GPE’s, e.g. mountain ranges, coasts, borders, planets, geocoordinates, bodies of water.  Also included in this category are named regions such as the Middle East, areas, neighborhoods, continents and regions of continents such as Eastern Europe.  

Unmarkable: Do not mark deictics or other non-proper nouns; here, there, everywhere, etc.  Directional modifiers such as western are not marked.
River: rivers

SGML:  <ENAMEX TYPE="LOCATION:RIVER"> </ENAMEX>

Lake_Sea_Ocean: Bay, Gulf, 

SGML:  <ENAMEX TYPE="LOCATION:LAKE_SEA_OCEAN"> </ENAMEX>

Border: named border regions
	the famous <Four Corners> intersection

SGML:  <ENAMEX TYPE="LOCATION:BORDER">  </ENAMEX> 

Region: Named areas larger than a city; Baltic, Bay Area, Bekka Valley, Benelux, Central America, East bloc, Indochina, Latin America, Long Island, Scandinavia, Silicon Valley, Urals,Western Hemisphere
	<New England>
	<Central America>
	the <Northeast>

SGML:  the <ENAMEX TYPE="LOCATION:REGION">  </ENAMEX>

Latitude-Longitude:

Continent: continents

SGML:  <ENAMEX TYPE="LOCATION:CONTINENT"> </ENAMEX>

Other:  Brighton Beach, Micro-rayon, Named districts of a city, School district
	<Earth>

SGML:  <ENAMEX TYPE="LOCATION:OTHER">  </ENAMEX>


Product Name
Markable:  Name of any product (but does NOT include the manufacturer), e.g., ‘Ford Taurus’ is an organization name followed by a product name.  This category covers any product name.  Product names of foods should be marked Product, not substance.  Products that are referred to using their manufacturer’s name (as in “I bought a Buick”), should be marked ORG name, not Product.  Services and financial plans are not markable.  

Unmarkable:  credit cards, checking accounts, CDs, credit plans
Weapon: Missiles, guns, bombs, etc.  Often what precedes a weapon-desc will be an org-name rather than a weapon name.  

SGML:  <ENAMEX TYPE="PRODUCT:WEAPON">  </ENAMEX>

Vehicle: Name of make, ex. (Subaru) Legacy

SGML:  <ENAMEX TYPE="PRODUCT:VEHICLE"> </ENAMEX>

Other:

SGML:  <ENAMEX TYPE="PRODUCT:OTHER"> </ENAMEX>


Product
Descriptor

Head of Descriptor
Markable:  Descriptions of weapons and vehicles only.  Cars, buses, machine guns, bombs, missiles, bullets, etc. 

SGML:  <ENAMEX TYPE="PRODUCT_DESC:SUBTYPE"> </ENAMEX>
 See Product Name for subtypes.


Date 
Markable:  Used to classify a reference to a date or period.  Ex:  November 2 2001, January, Monday, 60’s, 1940’s, several years.  Ex:  two months, 4 months, last week, last year, yesterday, tomorrow, spring, summer, fall, winter, fourth quarter, first quarter, today, etc.  Age also falls under this category, even when it’s a noun phrase referring to a person:  the 5-year-old, 5 years old, Jane Doe, 5, etc.  

Unmarkable: Dates that are part of rate expressions such as per/a day or per/a year should not be marked.
Date: answers the question “when” and includes absolute dates (March 1), relative dates that are not durations (last week, 3 years ago, 3 months ended Oct 1, first quarter), other examples: 
	<the quarter ending March 30>
	<June>
	<the previous quarter>
	<a year ago>
	<summer>
	<1960s>
	<20 years earlier>
	<a couple of years ago>
	<the quarter ending March 30>

Phrases with modifers like “before”, “after” “later” (these modifiers are included), 1990-1995, these days, today, yesterday, tomorrow, Tuesday, February 3, 2002

Conjoined dates, like “eight and ninth centuries,” or “23, 24, and 28 January,” or “May 3, 4, 10, and 11,” are markable as one entity.

SGML:  <TIMEX TYPE="DATE:DATE">  </TIMEX>

Duration: answers the question “how long” and includes a period of time (2 years, centuries, 16 weeks, less than 2 years, 6 months, 52-week).

SGML:  <TIMEX TYPE="DATE:DURATION">  </TIMEX>

Age:  
	Rudolph Agnew , <55 years old> 
	Neil Davenport, <47>

SGML:  <TIMEX TYPE="DATE:AGE">  </TIMEX>

Other: periodic dates (weekly, annually), terms indicating that something occurs repeatedly/habitually, (over) the years, every day

SGML:  <TIMEX TYPE="DATE:OTHER"> </TIMEX>

Unmarked: nowadays, now, past / future, for now / until now, earlier, all the time / at the same time


Time
Markable: Any time ending with A.M. or P.M.  The a.m. and p.m. must be tagged along with the numbers.   Other times of day (units smaller than a day) and time durations may be marked:  morning, noon, night, 3 hours.

SGML:  <TIMEX TYPE="TIME"> </TIMEX>

No Subtypes

Percent
Markable: Any percent.  Percent symbol or the actual word percent must be explicit and included in the extent.  Ex:  50%

SGML:  <NUMEX TYPE="PERCENT"> </NUMEX>
No Subtypes

Money
Markable: Any monetary value including all monetary denominations.  The monetary unit must be explicit and included in the tagging.  Examples:  50 yen, one million dollars, 17,000 pounds, $10.20.  Only values should be tagged, generic references to money should not.  For example, in “money invested in…”, there is no markup for “money”.  In rate expressions such as $ per unit, the unit should not be included in the extent.  For example, in “$3 per share”, the extent is <$3>.

SGML:  <NUMEX TYPE="MONEY”>  </NUMEX>
No Subtypes

Quantity
Markable: Used to classify measurements.  Ex:  4 miles, 4 grams, 4 degrees, 4 pounds, 4 ounces.
1d: length, distance (3 miles, 2 feet, etc.)
	<about 60 miles>
	<six-inch> concrete walls
	<100-mile> race

SGML:  <NUMEX TYPE="QUANTITY:1D">  </NUMEX>

2d: area (2 square feet)
	<250,000-square-foot> building
	a <132-acre> tract
	<more than four acres>

SGML:  <NUMEX TYPE="QUANTITY:2D">  </NUMEX>

3d: volume (3 cubic meters, etc.)
	a <1.5 million-gallon> saltwater aquarium
	<1,296,800 barrels> of crude oil

SGML:  <NUMEX TYPE="QUANTITY:3D">  </NUMEX>

Energy:
	<250-megawatt> phase of the project

SGML:  <NUMEX TYPE="QUANTITY:ENERGY">   </NUMEX>

Speed:
	<65 miles an hour>

SGML:  <NUMEX TYPE="QUANTITY:SPEED">  </NUMEX>

Temperature:
	<minus 321 degrees Fahrenheit>

SGML:  <NUMEX TYPE="QUANTITY:TEMPERATURE"> </NUMEX>

Acceleration:

Weight: 
	<30,537 metric tons>

SGML:  <NUMEX TYPE="QUANTITY:WEIGHT">  </NUMEX>

Other:
	<40 megabyte> hard drives
	<32-bit> chips

SGML:  <NUMEX TYPE="QUANTITY:OTHER">  </NUMEX>  


Ordinal
Markable:  All ordinal numbers.  Ex:  First, fourth, second

SGML:  <NUMEX TYPE="ORDINAL">  </NUMEX>
No Subtypes

Cardinal
Markable:  Numerals that provide a count or quantity and do not fall under a unit of measurement, money, percent, date or time.  Whole numbers, decimals and fractions are included.  

Ex:  half, hundreds, one-third, four, 4534.  Ex:  Nasdaq composite fill 1.39 to 451.37 - the decimals are cardinals because there is no monetary unit.  

Headless numerical phrases are also covered in this category.  Ex: ‘reducing employment from 18,000 to 16,000’.  

Numbers identifying list items should also be included.

SGML:  <NUMEX TYPE="CARDINAL"></NUMEX>
No Subtypes

Events

Name
Markable:  Named hurricanes, battles, wars, sports events, and other named events. Ex:  WWI, WWII, New York City Marathon, Oklahoma City bombing, etc.
War:
	the <Mexican Revolution>
	the <Vietnam War>

SGML:  <ENAMEX TYPE="EVENT:WAR">  </ENAMEX>

Hurricane:
	<Hurricane Hugo>

SGML: <ENAMEX TYPE="EVENT:HURRICANE">  </ENAMEX>

Other:  sports events, other named events
	the official <Indianapolis 500> announcer
	A <World Series> telecast
	<Senior Olympic games>
	the <Koreagate> scandal
	the <Tokyo Motor Show>

SGML: <ENAMEX TYPE="EVENT:OTHER">  </ENAMEX>


Plant

Name or Head of Descriptor
Markable:  Any plant, flower, tree, etc.  Black eyed Susan, Peonies, Joshua Tree, cactus, grass, etc.

	1989-90 <crop>
	hybrid <plants>
	an annual <herb> , <Plantago ovata>
	selling <flowers>
	pussy-willow <buds>
	The <grapevine>

SGML: <ENAMEX TYPE="PLANT">   </ENAMEX>
No Subtypes

Animal

Name or Head of Descriptor
Markable:  Any animal class or proper name of an animal, real or fictional.

	an elusive <sheep>
	a docile <butterfly>
	<animals> and beneficial <insects>
	his <cuckoo> 's egg
	Could be <Cuddles>, Mrs. Stinnett's <dog>.
	keep the <worm> from the apple

SGML:  <ENAMEX TYPE="ANIMAL">  </ENAMEX>
No Subtypes

Substance

Name or Head of Descriptor
Markable:  Any chemicals, elements, drugs, and foods.  Ex:  boron, penicillin, plutonium, bananas etc. Also other materials.  Both names and descriptors are markable.

This category in particular has been difficult to define and may overlap with other categories.   See subtypes section for possible ambiguities and more examples.

Food: food and drinks 
Possible ambiguities:
	Food vs Animal – Refence to the animal is markable as Animal but reference to the food is Substance:Food
	<beef> (marked Substance:Food)
	<cow> (marked Animal)

	Food vs Product - Product names for foods are markable as Product, not Substance, but references to the foods themselves are Substance:Food
	their breakfast <cereals> (marked Substance:Food)
	<Corn Flakes> (marked Product)

	Food vs Plant - 
	20 more <corn> than naturally pollinated plants (marked Plant)
	$ 2.15 a bushel for <corn>

	Other Examples
	<Champagne> and <dessert> followed
	<food> and <drinks> are banned
	duckling <mousseline>, lobster <consomme>, veal <mignon>       
	chocolate <terrine> with a raspberry <sauce>

SGML: <ENAMEX TYPE="SUBSTANCE:FOOD">  </ENAMEX>

Drug: medicines, narcotics, etc.
	approval for a new <drug>
	The <drug> , called <adenocard>
	the antibiotic <products>
	trade some of it for <cocaine>

 SGML: <ENAMEX TYPE="SUBSTANCE:DRUG">  </ENAMEX>

Nuclear: nuclear material
	<titanium>
	<plutonium>

SGML:  <ENAMEX TYPE="SUBSTANCE:NUCLEAR">   </ENAMEX>

Chemical: Copper, elements from the periodic table, gold, silver
	class of <asbestos> including <crocidolite>
	<copper>
	10 drop in <ozone>
	products derived from <salt> , such as <chlorine> and caustic <soda>
	<chemicals> 

SGML:  <ENAMEX TYPE="SUBSTANCE:CHEMICAL">  </ENAMEX>

Other:	blood, cholesterol, gas, lipoproteins, oil, pulp, water (not intended for drinking), tobacco, butterfat, dna, plastics, coal, ice, toxins, stones, diamonds, etc.

SGML:  <ENAMEX TYPE="SUBSTANCE:OTHER">  </ENAMEX>


Disease

Name or Head of Descriptor
Markable:  Any disease or medical condition, Ex:  Hodgkin’s disease, AIDS, cancer, malaria, acne.

	18 deaths from malignant "<mesothelioma>, lung <cancer> and <asbestosis>
	asbestos-related <diseases>
	help treat juvenile <diabetes>
	suffered from <malnutrition>
	died after a <stroke>
	suffering from <depression>

For this category, both names and heads of descriptors are markable.

SGML: <ENAMEX TYPE="DISEASE"> </ENAMEX>
No Subtypes

Work of Art

Name
Markable:  Titles of books, songs, and other creations.  Usually surrounded by quotation marks in the article (though the quotations are not included in the annotation).  Ex:  Empire Strikes Back, The Bible, Blue Moon.  Also includes awards.  Nobel Peace Prize, Emmy, Oscar. 

Only names are markable in this category.
Newspaper headlines should only be marked if they are referential.  In other words the headline of the article being annotated should not be marked but if in the body of the text here is a reference to an article, then it is markable as a work of art.

Book: 
	Dashiell Hammett, who wrote `` <The Maltese Falcon>

SGML:  <ENAMEX TYPE="WORK_OF_ART:BOOK">   </ENAMEX>

Play: 
Operas, musicals, plays

SGML:  <ENAMEX TYPE="WORK_OF_ART:PLAY">   </ENAMEX>

Song: 
Songs, instrumental music

SGML:  <ENAMEX TYPE="WORK_OF_ART:SONG">   </ENAMEX>

Painting:  
	Vincent van Gogh's `` <Irises>

SGML:  <ENAMEX TYPE="WORK_OF_ART:PAINTING">  </ENAMEX>

Sculpture:  
SGML:  <ENAMEX TYPE="WORK_OF_ART:SCULPTURE ">  </ENAMEX>

Other: TV shows, movies, awards 
	<Nobel Prize>
	a spin-off of `` <Cosby> 

SGML:  <ENAMEX TYPE="WORK_OF_ART:OTHER">   </ENAMEX>


Law

Name
Markable: Any document that has been made into a law.  Ex:  Bill of Rights, Equal Rights Amendments, IRS code 4.  The law must be named.  Legal documents and sections and chapters of legal documents. Treaties.

Examples:
	The <1988 trade act>
	the so-called special <301 provision> of the act
	the <Johnson Act>
	<Article II of the Constitution>
	the <Warsaw Pact>

SGML:  <ENAMEX TYPE="LAW">  </ENAMEX>
No Subtypes

Language

Name
Markable:  Any named language.

Examples:
	<Latin> liturgy
	novelistic tradition in <Arabic>
	speaking <Filipino>

SGML:  <ENAMEX TYPE="LANGUAGE">  </ENAMEX>
No Subtypes

Contact info
Markable: Address, email, phone #, URL.
See subtypes for examples.
Address: 
	<153 East 53rd St.>

SGML: <ENAMEX TYPE="CONTACT_INFO:ADDRESS">  </ENAMEX>

Email: 
SGML: <ENAMEX TYPE="CONTACT_INFO:EMAIL">  </ENAMEX>

Phone #:  
	<extension 29>
	<900-TELELAW>
	<800-462-9029>

SGML: <ENAMEX TYPE="CONTACT_INFO:PHONE"> </ENAMEX>

URL:  
	<www.usabanc.com>

SGML: <ENAMEX TYPE="CONTACT_INFO:url">  </ENAMEX>

Game

Name or
Head of Descriptor
Markable: Any named game such as a board game, any descriptor head, or any game type such as “baseball”.  

Examples:
	<tennis>
	<football>
	<poker>
	<Pachinko>, a kind of <pinball>
	<paddleball>
	<Pool>

SGML: <ENAMEX TYPE="GAME">  </ENAMEX>
No Subtypes


GENERAL GUIDELINES

1.	Markables/Extents

Mark head words for descriptor categories and full name for name name categories.  For time and number categories, mark the full time, date, or quantity expression, regardless of context.  The following examples should be marked in their entirety:  2 feet, 3 hours, 5 days ago, etc.

For descriptors we generally mark only one head word.  But there are cases in which the head is not sufficient.  The marked head should convey the meaning of the phrase so that it captures the difference between “vice president” and “president” for example. In these cases, the head should include the information necessary to distinguish its meaning.  So in the above example, <vice president> (rather than just “president”) is markable as the head of “vice president of accounting” because a vice president and a president are not equivalent.

In the case of conjunctions, mark one entity per head word.  However, in the case of range expressions for times, dates and numerical categories (like “between two and 3 weeks”), mark the entire expression.

Proper premodifiers are markable.  Some non-proper premodifiers are markable.  See section 2 for clarification.

2.	Non-proper Premodifiers
When a premodifier is a common noun, it should generally not be marked.  The exceptions are as follows:

	Occupational titles as in “president Bush”, “co-founder Gates”, “ senator Bentsen”, etc.  Do not include modifiers of those occupational premodifiers in the extent.  For example, in “Bankruptcy Judge Jones’, only the extents “Judge” and “Jones” are markable as person descriptor and person name, respectively.

	Nominalizations of clause-like structures whose object is a category of interest.  For example, “heroin smuggler” is a nominalization of X smuggles heroin.  In this case we would want to mark “heroin”.  On the other hand, “company funds” is not a nominalization of X funds company, so we do not mark “company”.

	Timex and Numex expressions in premodifier position should also be marked.

	Possessives should be marked in premodifier position:  in “the company’s meeting”, “the company” should be marked


3.	Unmarkables

Pronouns and pronominal elements like anaphoric “one”, “someone”, “everyone”, “others”, etc. are not markable.  

Headless entities with no clear reference like “the best” are not markable.  

Names embedded in atomic names are not markable.  Ex:  no markup for Dow Jones in “Dow Jones Industrial Average”

Generics and specifics are not distinguished.  All references should be marked.  For example, in “scientists often say…,” “scientists” is markable even though it is generic.
